# TRIM21 suppresses CHK1 activation by preferentially targeting CLASPIN for K63-linked ubiquitination

**DOI:** 10.1093/nar/gkac011

**Published:** 2022-01-20

**Authors:** Xuefei Zhu, Jingwei Xue, Xing Jiang, Yamin Gong, Congwen Gao, Ting Cao, Qian Li, Lulu Bai, Yuwei Li, Gaixia Xu, Bin Peng, Xingzhi Xu

**Affiliations:** Guangdong Key Laboratory for Genome Stability & Disease Prevention and Carson International Cancer Center and Marshall Laboratory of Biomedical Engineering, Shenzhen University School of Medicine, Shenzhen, Guangdong 518060, China; Key Laboratory of Optoelectronic Devices and Systems of Ministry of Education and Guangdong Province, College of Optoelectronic Engineering, Shenzhen University, Shenzhen, Guangdong 518060, China; Guangdong Key Laboratory for Genome Stability & Disease Prevention and Carson International Cancer Center and Marshall Laboratory of Biomedical Engineering, Shenzhen University School of Medicine, Shenzhen, Guangdong 518060, China; Guangdong Key Laboratory for Genome Stability & Disease Prevention and Carson International Cancer Center and Marshall Laboratory of Biomedical Engineering, Shenzhen University School of Medicine, Shenzhen, Guangdong 518060, China; Guangdong Key Laboratory for Genome Stability & Disease Prevention and Carson International Cancer Center and Marshall Laboratory of Biomedical Engineering, Shenzhen University School of Medicine, Shenzhen, Guangdong 518060, China; Shenzhen University-Friedrich Schiller Universität Jena Joint PhD Program in Biomedical Sciences, Shenzhen University School of Medicine, Shenzhen, Guangdong 518060, China; Guangdong Key Laboratory for Genome Stability & Disease Prevention and Carson International Cancer Center and Marshall Laboratory of Biomedical Engineering, Shenzhen University School of Medicine, Shenzhen, Guangdong 518060, China; Capital Normal University College of Life Science, Beijing 100048, China; Capital Normal University College of Life Science, Beijing 100048, China; Capital Normal University College of Life Science, Beijing 100048, China; Guangdong Key Laboratory for Genome Stability & Disease Prevention and Carson International Cancer Center and Marshall Laboratory of Biomedical Engineering, Shenzhen University School of Medicine, Shenzhen, Guangdong 518060, China; Key Laboratory of Optoelectronic Devices and Systems of Ministry of Education and Guangdong Province, College of Optoelectronic Engineering, Shenzhen University, Shenzhen, Guangdong 518060, China; Guangdong Key Laboratory for Genome Stability & Disease Prevention and Carson International Cancer Center and Marshall Laboratory of Biomedical Engineering, Shenzhen University School of Medicine, Shenzhen, Guangdong 518060, China; Guangdong Key Laboratory for Genome Stability & Disease Prevention and Carson International Cancer Center and Marshall Laboratory of Biomedical Engineering, Shenzhen University School of Medicine, Shenzhen, Guangdong 518060, China; Shenzhen University-Friedrich Schiller Universität Jena Joint PhD Program in Biomedical Sciences, Shenzhen University School of Medicine, Shenzhen, Guangdong 518060, China

## Abstract

Expression of the E3 ligase TRIM21 is increased in a broad spectrum of cancers; however, the functionally relevant molecular pathway targeted by TRIM21 overexpression remains largely unknown. Here, we show that TRIM21 directly interacts with and ubiquitinates CLASPIN, a mediator for ATR-dependent CHK1 activation. TRIM21-mediated K63-linked ubiquitination of CLASPIN counteracts the K6-linked ubiquitination of CLASPIN which is essential for its interaction with TIPIN and subsequent chromatin loading. We further show that overexpression of TRIM21, but not a TRIM21 catalytically inactive mutant, compromises CHK1 activation, leading to replication fork instability and tumorigenesis. Our findings demonstrate that TRIM21 suppresses CHK1 activation by preferentially targeting CLASPIN for K63-linked ubiquitination, providing a potential target for cancer therapy.

## INTRODUCTION

Genomic DNA is constantly exposed to endogenous and exogenous insults and thus its stability and integrity is threatened. Accumulation of DNA damage due to these insults is linked to cancer etiology and progression (1). Replicating DNA is particularly sensitive to these insults, which may result in DNA aberrations such as DNA secondary structures, repetitive sequences, DNA–RNA hybrids, or DNA damage, and the progression of replication forks are stalled once they encounter those aberrations (2,3). A stalled replication fork is an unstable structure, which is predisposed to collapse, generating deleterious DNA double strand breaks (DSBs) if not stabilized and repaired (3). Stalled replication forks efficiently activate ATR-CHK1 checkpoint signaling to stabilize stalled forks and halt cell cycle progression, assuring accurate duplication and passage of genomic information (2–5). Single strand DNA (ssDNA) generated at stalled replication forks due to the uncoupling of CMG helicases and replicative polymerases, is rapidly bound by the replication protein A (RPA) complex (RPA1, RPA2 and RPA3); this serves as a platform for ATR-ATRIP recruitment through the interaction between ATRIP and RPA (6,7). The full activation of ATR also requires the coordination of RAD17, the 9–1–1 complex and TOPBP1 (8,9). RPA also recruits the TIMELESS–TIPIN complex through an interaction between RPA2 and TIPIN, which further interacts with and stabilizes CLASPIN on RPA-coated ssDNA where CLASPIN becomes phosphorylated in an ATR-dependent manner (10). CLASPIN phosphorylation is required for its interaction with CHK1 and thereby ATR-mediated CHK1 phosphorylation and activation (11,12).

As an adaptor protein mediating ATR-dependent CHK1 phosphorylation and activation, CLASPIN expression is strictly regulated throughout the cell cycle, with relatively low expression at G1 phase, high expression at S/G2 phase, and back to low level expression at M phase (13). The dynamic expression levels of CLASPIN are regulated by the E3 ligase APC^Cdh1^ complex at G1 phase, which can be antagonized by the deubiquitinase USP28 at S/G2 phase (14). At M phase, the expression levels of CLASPIN are regulated by the E3 ligase SCF^βTrCP^ complex, which can be antagonized by USP7 (15–18). In addition, several other deubiquitinases have also been reported to regulate the stability of CLASPIN, such as USP29, USP9X and USP20 (19–22). BRCA1 also regulates CLASPIN ubiquitination at its N-terminus, which is not responsible for CLASPIN turnover but instead promotes its loading on chromatin where it mediates ATR-dependent CHK1 activation (10,23,24). This finding indicates the potential involvement of non-degradation-related ubiquitination on CLASPIN in mediating ATR-CHK1 activation.

Some tripartite motif (TRIM) proteins positively or negatively regulate carcinogenesis via the DNA damage response pathway (25,26). TRIM proteins constitute a RING type E3 ligase subfamily incorporating >70 members, which are characterized by regular sequence of RING domain, one or two B-boxes and a coiled-coil region from N-terminus to C-terminus, with several exceptions without the RING domain (25,27). TRIM21 is a tripartite motif (TRIM)-containing protein, often found overexpressed in patients suffering from autoimmune diseases. This protein participates in a series of pathways such as cytokinesis and redox regulation (28–30). Previous studies have found that TRIM21 is upregulated in cancers (31,32). Given the relationship between TRIM proteins and DNA damage pathways, we hypothesized this mechanism might be linked to replication fork stalling.

By performing a series of *in vitro* and *in vivo* analyses, we found that TRIM21 serves as a novel E3 ligase of CLASPIN in response to DNA replication stress. TRIM21-mediated CLASPIN K63-linked ubiquitination counteracts its K6-linked ubiquitination, repressing chromatin loading of CLASPIN and activation of CHK1 upon replication stress. Therefore, TRIM21 overexpression compromises the stability of stalled replication forks and promotes tumorigenesis.

## MATERIALS AND METHODS

### Cell culture and transfection

U2OS, HeLa, HEK293T, HCT116 and U87 cell lines were obtained from the American Type Culture Collection. All cell lines were cultured with high-glucose Dulbecco's modified Eagle's medium (HyClone) supplemented with 10% fetal bovine serum (PAN-Biotech) and penicillin-streptomycin (HyClone), at 37°C with 5% CO_2_. Cell transfection was performed using 1 mg/ml Polyethylenimine, Linear (PEI, polysciences) following the manufacturer's protocol.

### Plasmid constructs

Human full length CLASPIN, TRIM21, TIPIN and ubiquitin cDNAs were sub-cloned into a pcDNA3.0 expression vector with an HA or FLAG N-terminal epitope; TRIM21 and ubiquitin cDNAs were also cloned into pcDNA3.1-MYC or pCMV-MYC vectors, respectively. pET28a and pGEX-4T-1 bacterial expression vectors were used for either the HIS or GST tags. TRIM21 was cloned into pET28a vector. CLASPIN 1–330aa, 301–630aa, 601–930aa and 901–1339aa fragments were cloned into pGEX4T-1 vector. HA-ubiquitin K63 only was also cloned into pGEX4T-1 vector (GST tag was cleavable by thrombin). Catalytically inactive mutant TRIM21CA (C16A/C31A/H33W), ubiquitin mutants (K6 only, K63 only and K48 only) and CLASPIN mutants (KR mutant or deletion mutants) were generated using the Mut Express II Fast Mutagenesis Kit V2 (Vazyme).

### Antibodies and reagents

Polyclonal antibodies for anti-CLASPIN (A300–266A), anti-TRIM21 (A302-519A), Anti-CHK1(A300-298A), anti-HA (A190–208A) and anti-MYC (A190–205A) were purchased from Bethyl Laboratories. Antibodies for anti-TRIM21(ab201628) and anti-BrdU (ab6326) were purchased from Abcam. Antibodies for anti-pCHK1^S345^(#2348) and anti-H3(#9715) were purchased from Cell Signaling Technology. Anti-Actin(A5441) and anti-Flag (F1804) antibodies were from Sigma. Anti-GST (M209-3) and anti-His(D291-3) antibodies were from MBL. Anti-BrdU (BD347580) antibody was from BD Biosciences. Antibodies for anti-LaminB1 (A1910) and anti-β-Tubulin (AC008) were purchased from ABclonal. Protein A Sepharose™ CL-4B, Glutathione Sepharose™ 4B and Ni Sepharose™ 6 Fast Flow were purchased from GE Healthcare. Anti-FLAG M2 affinity gel (A2220), hydroxyurea, aphidicolin, camptothecin, CldU and IdU were purchased from Sigma. Recombinant human UBE1 (E-305), UbcH5b/UBE2D2 (E2-622), and HA-ubiquitin (U-110) were purchased from BostonBiochem. Thrombin (T8021) was purchased from Solarbio.

### RNA interference

The introduction of small interfering RNA (siRNA) into U2OS, HeLa or HEK293T cells was carried out with RNAiMAX (Invitrogen) following the manufacturer's protocol. The siRNAs directed against TRIM21 or CLASPIN were synthesized by Genepharma. The siRNA sequences were as follows: siNC: UUCUCCGAACGUGUCACGU; siTRIM21-1#: GGAAGUCACUUCACCAUCA; siTRIM21-2#: GUGAAGCAGCCUCCUUAUA; the sequence of the siRNA directed against CLASPIN was described previously (33).

### Immunoprecipitation and pull-down assay

Immunoprecipitation and pull-down assay were performed with the indicated proteins as described previously (34). In brief, HEK293T cells transfected with the indicated plasmids were lysed in NETN buffer (100 mM NaCl, 1 mM EDTA, 20 mM Tris–HCl [pH 8.0] and 0.5% NP-40) containing a protease inhibitor cocktail (Roche) for 30 min at 4°C and then centrifuged. For endogenous immunoprecipitation, cells lysates were incubated with an anti-CLASPIN antibody for 4 h and then with protein-A beads (2 mg/ml) for 1 h, followed by extensive washes with NETN buffer for 10 min × 3 times at 4°C. For exogenous immunoprecipitation, cell lysates were incubated with M2 beads for 4 h, followed by extensive washes with NETN buffer at 4°C. Bead-bound proteins were denatured in 2× sample buffer (62.5 mM Tris–HCl [pH 6.8], 2% SDS, 20 mM DTT and 10% glycerol) at 100°C for 5 min, and then resolved by SDS-PAGE and examined by immunoblotting with the indicated antibodies.

For pull-down assays, bacterially purified HIS-TRIM21 and GST/GST-CLASPIN 301–630aa immobilized on Glutathione Sepharose 4B beads were incubated for 4 h at 4°C. Then, bead-bound proteins were denatured and HIS-TRIM21 was examined by immunoblotting, while GST/GST-CLASPIN 301–630aa was visualized by Ponceau S staining.

### Cell-cycle distribution and S phase synchronization analysis

Cells were labeled with 20 μM BrdU for 30 min, harvested by trypsinization and washed twice with cold PBS before fixed with ice-cold 70% ethanol for 16 h. Cells were then washed twice with 1% BSA and permeabilized with 0.5% Triton X-100 for 20 min, denatured with 2 M HCl for 30 min, and followed by neutralization with 0.1 M Na_2_B_4_O_7_ for 10 min. After being washed twice with 1% BSA, cells were incubated with anti-BrdU (BD347580) antibody for 1 h at 37°C, and washed with PBST (0.1% Tween-20) for three times. FITC-conjugated secondary antibody incubation was performed for 1 h at 37°C. After washing with PBST for three times, cells were resuspended in propidium iodide/RNase and incubated for 30 min before the cell-cycle distribution was analyzed by flow cytometry. S-phase synchronization by double thymidine blocks was performed as described previously (35). In brief, cells were treated with 2 mM thymidine for 17 h and released in fresh medium for another 8 h. 2 h after release from the second block with 2 mM thymidine for 18 h, cells were pulse labeled with 20 μM BrdU for 30 min, and subjected to staining with anti-BrdU antibody and PI as described above, followed by analysis with flow cytometry.

### Chromatin fractionation

Chromatin fractionation was performed as described previously (36). In brief, HeLa cells were transfected with a negative control or different TRIM21 specific siRNAs, followed by double thymidine block and release to synchronize cells in S phase as described above. Cells were then harvested by trypsinization and washed twice with cold PBS. Collected cells were resuspended in cold Buffer A (10 mM HEPES [pH 7.9], 10 mM KCl, 1.5 mM MgCl_2_, 0.34 M sucrose, 10% glycerol, 1 mM dithiothreitol and protease inhibitor) with a final concentration of 0.1% Triton X-100, and incubated on ice for 5 min. Nuclei were collected by centrifugation (1500 × g, 4 min, 4°C) and washed once with buffer A, followed by lysis with buffer B (3 mM EDTA, 0.2 mM EGTA, 1 mM dithiothreitol, and protease inhibitor). After incubation on ice for 10 min, chromatin was separated by centrifugation (2000 × g, 4 min, 4°C), washed once with buffer B and collected by centrifugation (13 000 × g, 1 min, 4°C).

### DNA fiber assay

HeLa cells with the indicated TRIM21 background were sequentially labeled with 40 μM CldU and 100 μM IdU for 30 min each at 37°C, followed by treatment with 5 mM HU and 5 μM APH for 2 h to block replication progress. Then, the cells were dissociated by trypsinization and mixed with unlabeled cells to perform a DNA fiber assay, as described previously (37).

### Metaphase spread assay

Metaphase spread assay was performed as previously described (38). Briefly, HeLa cells stably overexpressing empty vector, TRIM21 or TRIM21CA mutant were treated with 0.4 μg/ml colchicine for 4 h before being harvested. After hypotonic treatment with 0.056 M KCl and fixation with methanol/acetic acid (volume ratio of 3:1), the chromosome spreads were prepared and stained with Giemsa and then images were captured under a DragonFly confocal imaging system (Andor). For each experiment, >1500 metaphase chromosomes were analyzed.

### Ubiquitination assay *in vitro* and *in vivo*

A modified *in vitro* ubiquitination assay was performed as described previously (39) with some modifications. Recombinant human UBE1 (100 nM), UbcH5b/UBE2D2 (1 μM), and HA-ubiquitin or HA-ubiquitin K63 only (10 μM) were mixed in ubiquitination buffer (25 mM Tris–HCl [pH 8.0], 100 mM NaCl, 1 mM DTT, 2.5 mM ATP, 4 mM MgCl_2_) with the indicated final concentration. The recombinant human GST-CLASPIN 301–630aa and HIS-TRIM21 (or HIS-TRIM21 CA mutant) peptides expressed in *Escherichia coli* were also included to a final volume of 50 μl. The reaction mixtures were incubated for 60 min at 30°C followed by GST-pulldown.

For the *in vivo* ubiquitination assay, HEK293T cells were transfected with the indicated siRNA or expression vectors, and incubated for 24–48 h before being harvested. The cells were lysed in 62.5 mM Tris–HCl (pH6.8), 2% SDS and 10% glycerol with protease inhibitor cocktail, boiled for 10 min and centrifuged to remove cell debris. Then, the cell lysates were diluted 10× with NETN buffer (20 mM Tris–HCl pH8.0, 100 mM NaCl, 1 mM EDTA, 0.5% NP40) and subjected to immunoprecipitation with the indicated antibodies, as described.

### Nude mice tumor xenograft

Tumor xenograft experiments were performed as described previously(21). In brief, 14 male BALB/c nude mice (6–8 weeks old with body weight of 16–18 g, purchased from Beijing Vital River Laboratory Animal Technology Co., Ltd.) were randomly assigned to two groups. Then, 6 × 10^6^ HCT116 cells overexpressing empty vector or TRIM21 were injected subcutaneously into the right lower flanks of mice. The tumor volume was measured in two dimensions starting from day 3 after implantation and ending at day 23, and was calculated as 0.5 × length × width × width. Tumor volumes were analyzed by statistical analysis *t*-test, and shown as mean ± SEM. Animal experiments were performed according to protocols approved by the Committee on the Use of Live Animals in Teaching and Research of Shenzhen University.

## RESULTS

### Depletion of TRIM21 accelerated CHK1 activation after replication stress

**Figure 1.**
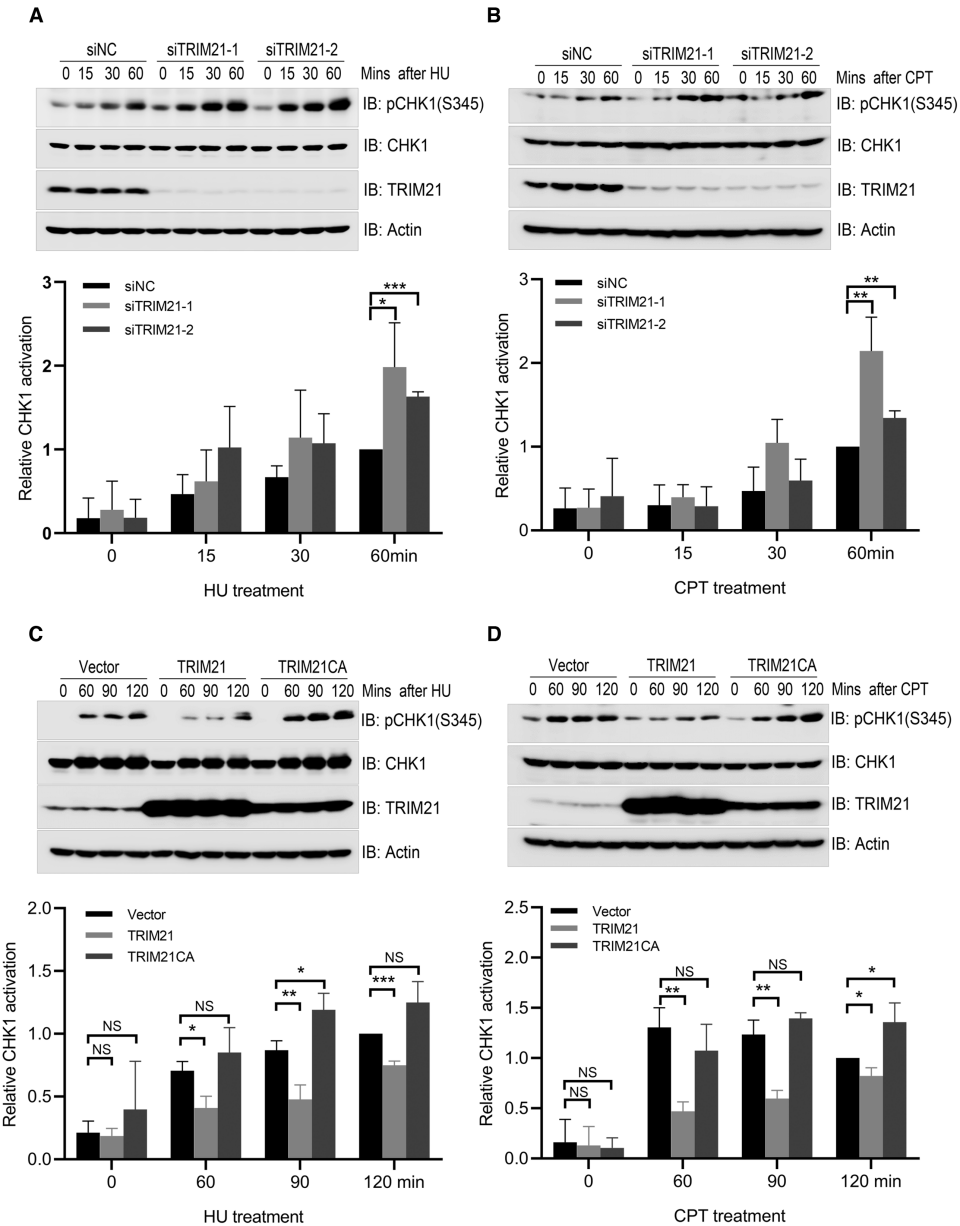
TRIM21 negatively regulated CHK1 activation upon replication stress. (**A**) HeLa cells were transfected with a negative control siRNA (siNC) or siRNA targeting 3’UTR of TRIM21 (siTRIM21-1 and siTRIM21-2) for 48 h. Transfectants were treated with 2 mM HU for the indicated time and total cell lysates were harvested for immunoblotting with antibodies as indicated. Statistical analysis (*t*-test) of signal intensity from three independent experiments was shown in the lower panel. *P < 0.05; ***P < 0.001. (**B**) HeLa cells were treated as described in (A) except that HU treatment was replaced with CPT (1 μM) treatment. **P < 0.01. (**C**) HeLa cells overexpressing TRIM21 or catalytically inactive mutant TRIM21CA were treated with 2 mM HU for the indicated time before total cell lysates were extracted for immunoblotting with antibodies as indicated. Statistical analysis (*t*-test) of signal intensity from three independent experiments was shown in the lower panel. *P < 0.05; **P < 0.01; ***P < 0.001. (**D**) HeLa cells overexpressing TRIM21 or TRIM21CA were treated with 1 μM CPT for the indicated time before total cell lysates were extracted for immunoblotting with antibodies as indicated. *P < 0.05; **P < 0.01.

The GEPIA is an analytical platform for RNA sequencing expression data of tumors and normal samples from the TCGA and the GTEx projects (40). We first analyzed the GEPIA to determine TRIM21 expression profile across different tumors. We found that TRIM21 expression levels are potentially elevated in a wide spectrum of tumors (Supplementary Figure S1), including glioma, melanoma, pancreatic adenocarcinoma, testicular germ cell tumors, acute myeloid leukemia, cholangio carcinoma and colorectum adenocarcinoma. This finding suggests that TRIM21 might have a role in tumorigenesis. High TRIM21 expression levels in glioma cells confer resistance to temozolomide by suppressing the p53–p21 pathway(32). Temozolomide is a DNA alkylating agent used for brain tumor chemotherapy, inducing cell senescence via activation of the ATR-CHK1, p53-p21 and NF-κB pathways(41). This finding prompted us to explore whether TRIM21 has a role in the regulation of ATR-CHK1 activation. As reported previously (42–44), we confirmed that TRIM21 was present in the nuclear fraction (Supplementary Figure S2A). We detected an increase of CHK1 activation, as measured by CHK1 phosphorylation at Ser345, this increase was induced by temozolomide treatment in a TRIM21 knockdown glioblastoma U87 cell line (Supplementary Figure S2B). We further examined if modulation of TRIM21 expression had any impact on CHK1 activation upon hydroxyurea (HU, inhibitor of class I ribonucleotide reductase) or camptothecin (CPT, inhibitor of DNA topoisomerase I) treatment. Indeed, inhibiting TRIM21 expression with siRNA resulted in an enhancement of CHK1 activation induced by HU or CPT treatment in HeLa cells (Figure 1A, B, and Supplementary Figure S2E) and U2OS cells (Supplementary Figures S2C, S2D and S2F). In HeLa cells, overexpression of wild-type TRIM21, but not the catalytically inactive mutant TRIM21 CA, resulted in compromised HU or CPT-induced CHK1 activation (Figure 1C and D). These data indicate that the E3 ligase activity of TRIM21 negatively regulates CHK1 activation induced by replication stress.

### TRIM21 ubiquitinated CLASPIN and repressed CLASPIN-dependent CHK1 activation

**Figure 2.**
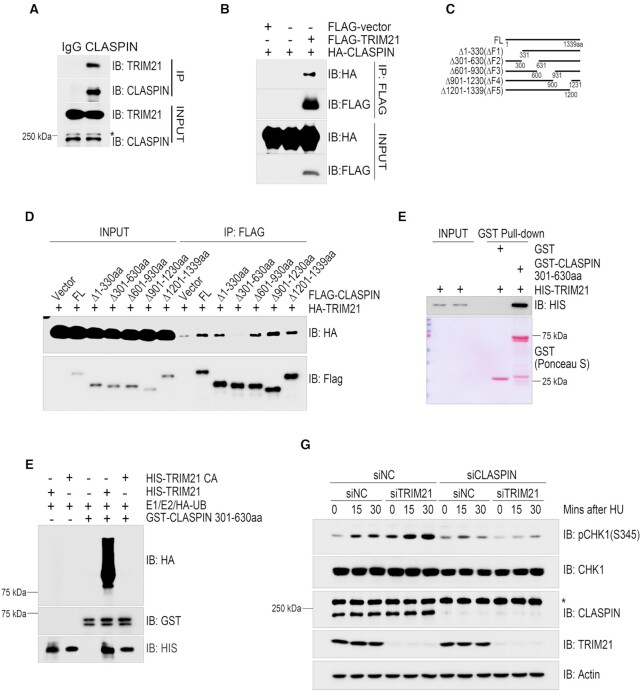
TRIM21 ubiquitinated CLASPIN and regulated CLASPIN dependent CHK1 activation. (**A**) HEK293T cells were lysed and incubated with protein A agarose conjugated with normal rabbit IgG or anti-CLASPIN antibody for immunoprecipitation, followed by immunoblotting with the indicated antibodies. *, non-specific signal. (**B**) HEK293T cells co-transfected with HA-CLASPIN and FLAG-TRIM21 were lysed and incubated with anti-FLAG M2 agarose, the immunoprecipitates were then examined by immunoblotting with antibodies against HA and FLAG. (**C**) Schematic of the CLASPIN deletion mutants: F1:1–330aa, F2:301–630aa, F3:601–930aa, F4:901–1230aa, F5:1201–1339aa. (**D**) Whole cell lysates extracted from HEK293T cells co-transfected with HA-TRIM21 and wildtype or mutant FLAG-CLASPIN were incubated with anti-FLAG M2 agarose for immunoprecipitation, followed by immunoblotting with the indicated antibodies. (**E**) Bacterially-purified HIS-TRIM21 and GST-CLASPIN301-630aa/GST peptides immobilized on Glutathione Sepharose 4B beads were mixed and incubated for 4 h. The HIS-TRIM21 complex was examined by immunoblotting. GST-CLASPIN301-630aa/GST were directly visualized by Ponceau S staining. (**F**) *In vitro* ubiquitination assays were performed by incubating HIS-TRIM21 or its E3 ligase inactive mutant and GST-CLASPIN301-630aa in the presence of E1, E2 and HA-ubiquitin at 30°C for 1 h, followed by GST-pulldown and analysis by immunoblotting with the indicated antibodies. (**G**) HeLa cells were transfected with a negative control siRNA or an siRNA targeting TRIM21 or CLASPIN as indicated for 48 hours. Then, whole cell lysates were collected after being treated with 2 mM HU or a mock treatment for the indicated times. CHK1 activation was examined by immunoblotting with an antibody specifically recognizing phosphorylated CHK1 at Ser345. The expression levels of CLASPIN, TRIM21 and CHK1 were also examined. *Non-specific signal. Actin: loading control.

We next sought to determine the substrate for TRIM21 to suppress CHK1 activation. We found that endogenous TRIM21 was present in the CLASPIN immunocomplex (Figure 2A) and HA-CLASPIN was present in the FLAG-TRIM21 immunocomplex when both were transiently expressed in HEK293T cells (Figure 2B). To map the essential region in CLASPIN required for its interaction with TRIM21, we generated a series of overlapping deletion mutants in the context of full-length CLASPIN (Figure 2C). Co-immunoprecipitation assays revealed that the FLAG-tagged CLASPIN ΔF2 mutant, in which the region between 301 and 630aa was deleted, failed to co-precipitate with HA-TRIM21 when both were co-expressed in HEK293T cells (Figure 2D). We further narrowed down the deletion to Δ331–400, Δ401–500 or Δ501–600, and did not detect an impaired interaction between TRIM21 and these CLASPIN mutants, indicating this interaction could be mediated by multi-sites on 331–600aa (Supplementary Figure S3). We next investigated if TRIM21 directly interacted with CLASPIN. GST pulldown assays found that a bacterially produced GST fusion of the CLASPIN F2 region (301–630aa) was sufficient to pull down bacterially-produced HIS-TRIM21 (Figure 2E).

Given the direct interaction demonstrated between TRIM21 and CLASPIN, we reasoned that CLASPIN could be a TRIM21 substrate. Indeed, *in vitro* ubiquitination assays found that bacterially produced wild type TRIM21, but not catalytically inactive mutant TRIM21 CA, ubiquitinated bacterially produced GST-CLASPIN (301–630aa) (Figure 2F). To demonstrate that TRIM21 mediates the suppression of CHK1 activation by targeting CLASPIN, we compared the CHK1 activation dynamics in TRIM21-depleted, CLASPIN-depleted, and TRIM21/CLASPIN-depleted cells. We found that inhibition of CLASPIN expression decreased CHK1 activation, while inhibition of TRIM21 expression enhanced CHK1 activation. Double inhibition of TRIM21 and CLASPIN expression did not lead to obvious CHK1 activation (Figure 2G). Together, these data demonstrate that TRIM21 suppresses CHK1 activation through direct interaction with and potential ubiquitination of CLASPIN.

### TRIM21 promoted K63-linked ubiquitination of CLASPIN and counteracted its K6-linked ubiquitination

To test the TRIM21-mediated ubiquitin linkage type on CLASPIN, we used HEK293T cells and initially examined if TRIM21 targeted CLASPIN for K48-linked ubiquitination. This modification marks a substrate for proteasome degradation (45). Inhibiting TRIM21 expression with two independent siRNA oligos did not change the K48-linked ubiquitination levels of CLASPIN (Supplementary Figure S4A). This observation was supported by the fact that inhibiting TRIM21 expression did not have an obvious impact on CLASPIN protein levels (Supplementary Figure S4B).

**Figure 3.**
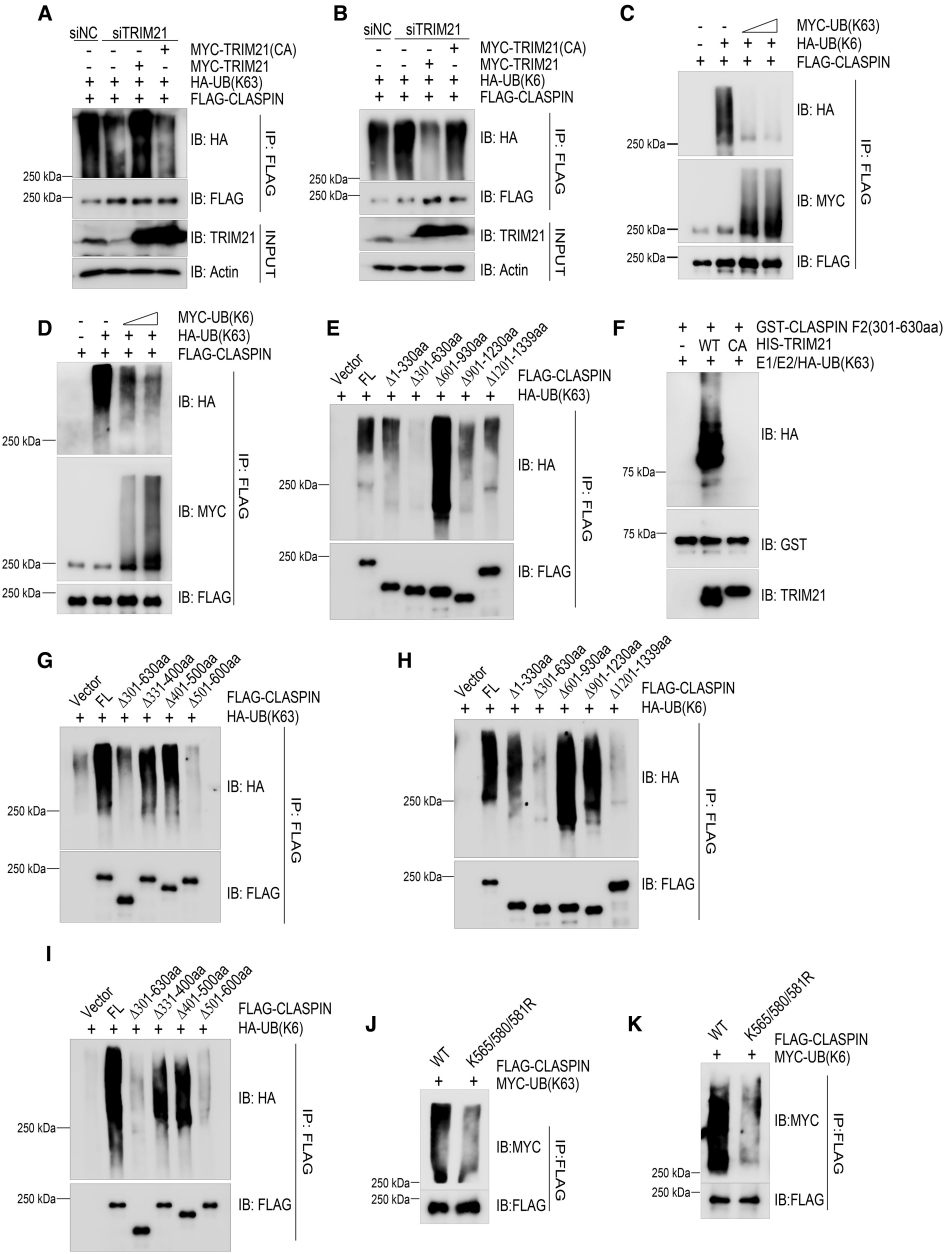
TRIM21 ubiquitinated CLASPIN with K63 linkage that counteracted its K6-linkage ubiquitination. (A, B) HEK293T cells were transfected with a negative control or a TRIM21-specific siRNA for 24 h. Then, the cells were co-transfected with FLAG-CLASPIN and HA-ubiquitin K63-only (as in **A**) or HA-ubiquitin K6-only (as in **B**) together with MYC-TRIM21 or MYC-TRIM21 E3 ligase inactive mutant as indicated. After 24 h, all the cell lines were lysed and subjected to de-naturing immunoprecipitation using anti-FLAG M2 agarose. The immunoprecipitates were examined by immunoblotting with the indicated antibodies. (C, D) HEK293T cells were transfected with FLAG-CLASPIN, and HA-ubiquitin K6-only along with increasing doses of MYC-ubiquitin K63-only expressing plasmids (as in **C**) or HA-ubiquitin K63-only along with increasing doses of MYC-ubiquitin K6-only expressing plasmids (as in **D**). After 24 h, the cells were lysed and analyzed by de-naturing immunoprecipitation using anti-FLAG M2 agarose, followed by immunoblotting with the indicated antibodies. (**E**) HEK293T cells were co-transfected with HA-ubiquitin K63-only and full-length FLAG-CLASPIN or its deletion mutants, and then analyzed by de-naturing immunoprecipitation and immunoblotting with the indicated antibodies. (**F**) *In vitro* ubiquitination assays were performed by incubating HIS-TRIM21 or TRIM21CA with GST-CLASPIN 301–630aa in the presence of E1, E2 and HA-ubiquitin-K63 only (HA-UB(K63)) at 30°C for 1 h, followed by GST-pulldown and analysis by immunoblotting with the indicated antibodies. (**G**) K63-linked ubiquitination of wildtype CLASPIN and its deletion mutants was examined as described in (E). (**H**) HEK293T cells were co-transfected with HA-ubiquitin K6-only and full-length FLAG-CLASPIN or its deletion mutants, and analyzed by denaturing immunoprecipitation and immunoblotting with the indicated antibodies. (**I**) HEK293T cells were transfected with the indicated plasmids and analyzed by de-naturing immunoprecipitation and immunoblotting with the indicated antibodies. (**J**, **K**) HEK293T cells were co-transfected with MYC-ubiquitin K63-only (as in J) or MYC-ubiquitin K6-only (as in K) and wildtype FLAG-CLASPIN or its K565/580/581R mutant. The samples were analyzed by de-naturing immunoprecipitation and immunoblotting with the indicated antibodies.

We next determined if TRIM21 ubiquitinated CLASPIN via K63 or K6 linkages, both of which enable substrates to participate in cell signaling and/or protein-protein interactions. Overexpression of TRIM21 promoted the K63-linkage type of ubiquitination on CLASPIN (Supplementary Figure S5), while inhibiting TRIM21 expression reduced the K63-linkage type of ubiquitination on CLASPIN, and re-expression of wild type TRIM21, but not the catalytically inactive mutant TRIM21 CA, restored K63-linked ubiquitination of CLASPIN (Figure 3A). Conversely, inhibiting TRIM21 expression enhanced K6-linked ubiquitination on CLASPIN, while re-expressing wild type TRIM21, but not the catalytically inactive mutant TRIM21 CA, suppressed the K6-linked ubiquitination of CLASPIN (Figure 3B).

These findings indicate that TRIM21 ubiquitinates CLASPIN with K63-linkage type, and this ubiquitination might counteract K6-linked ubiquitination of CLASPIN. Indeed, when we co-expressed FLAG-CLASPIN, HA-UB(K6), and increasing amounts of MYC-UB(K63) in HEK293T cells, the K63-linked ubiquitination of CLASPIN increased in a dose-dependent manner, while the K6-linked ubiquitination of CLASPIN decreased in a dose-dependent manner (Figure 3C). Conversely, increasing ubiquitination of CLASPIN with the K6 linkage type correlated with decreasing ubiquitination with the K63 linkage type (Figure 3D). Together, these results support that TRIM21 promotes K63-linked ubiquitination of CLASPIN, which counteracts its K6-linked ubiquitination.

### Homeostasis of K63/K6-linked ubiquitination of CLASPIN regulated by TRIM21 falls in the region of 501-600aa of CLASPIN

To understand how TRIM21 regulates the homeostasis of K63/K6-linked ubiquitination of CLASPIN, we sought to determine the essential region/site(s) of ubiquitination on CLASPIN. Harnessing a series of overlapping deletion mutants of CLASPIN (illustrated in Figure 2C), *in vivo* ubiquitination assays revealed that the ΔF2 (301–630aa) mutant lost K63-linked ubiquitination (Figure 3E), indicating the major K63-linked ubiquitination site(s) resides within the region of 301–630aa. Examination of the total ubiquitination level of CLASPIN deletion mutants showed that total ubiquitination modification mainly located at N-terminal 1–330aa (Supplementary Figure S6). While *in vitro* ubiquitination assays revealed that TRIM21, but not the catalytically inactive mutant TRIM21(CA), directly ubiquitinate F2 fragment with K63-linkage (Figure 3F). In addition to F2 fragment, we also detected a weak HA signal on F3 fragment after long exposure and a strong HA signal on F4 + F5 fragment, which indicated mono/oligo-ubiquitination without obvious polyubiquitination (Supplementary Figure S7A). Further mapping by *in vivo* ubiquitination assay uncovered that deletion of the region of 501–600 aa diminished its K63 ubiquitination to similar levels to that of the ΔF2 mutant (Figure 3G). On the other hand, both the ΔF2 mutant and ΔF5 mutant (deletion of the region of 1201–1399aa) lost K6-linked ubiquitination (Figure 3H). However, inhibition of TRIM21 expression by two independent siRNA oligos resulted in an increase of K6-linked ubiquitination of the ΔF5 mutant but not the ΔF2 mutant (Supplementary Figures S7B and S7C), indicating that the major K6-linked ubiquitination of CLASPIN regulated by TRIM21 resides within the region of 301–630aa. This finding is supported by the counteraction between K6/K63 linkage ubiquitination of the CLASPIN F2(301–630aa) fragment (Supplementary Figure S7D). We narrowed down the sites further to the region of 501–600 aa (Figure 3I), which is enriched with lysine sites. Finally, we showed that the K565/580/581R mutants compromised both K63-linked and K6-linked ubiquitination (Figure 3J, K, and Supplementary Figures S7E-S7G), indicating that these two modifications counteracted with each other by competing lysine sites. Together, these data support that TRIM21 regulates the homeostasis of K63/K6-linked ubiquitination located in the region of 501–600 aa of CLASPIN.

### TRIM21 negatively regulated chromatin loading of CLASPIN

**Figure 4.**
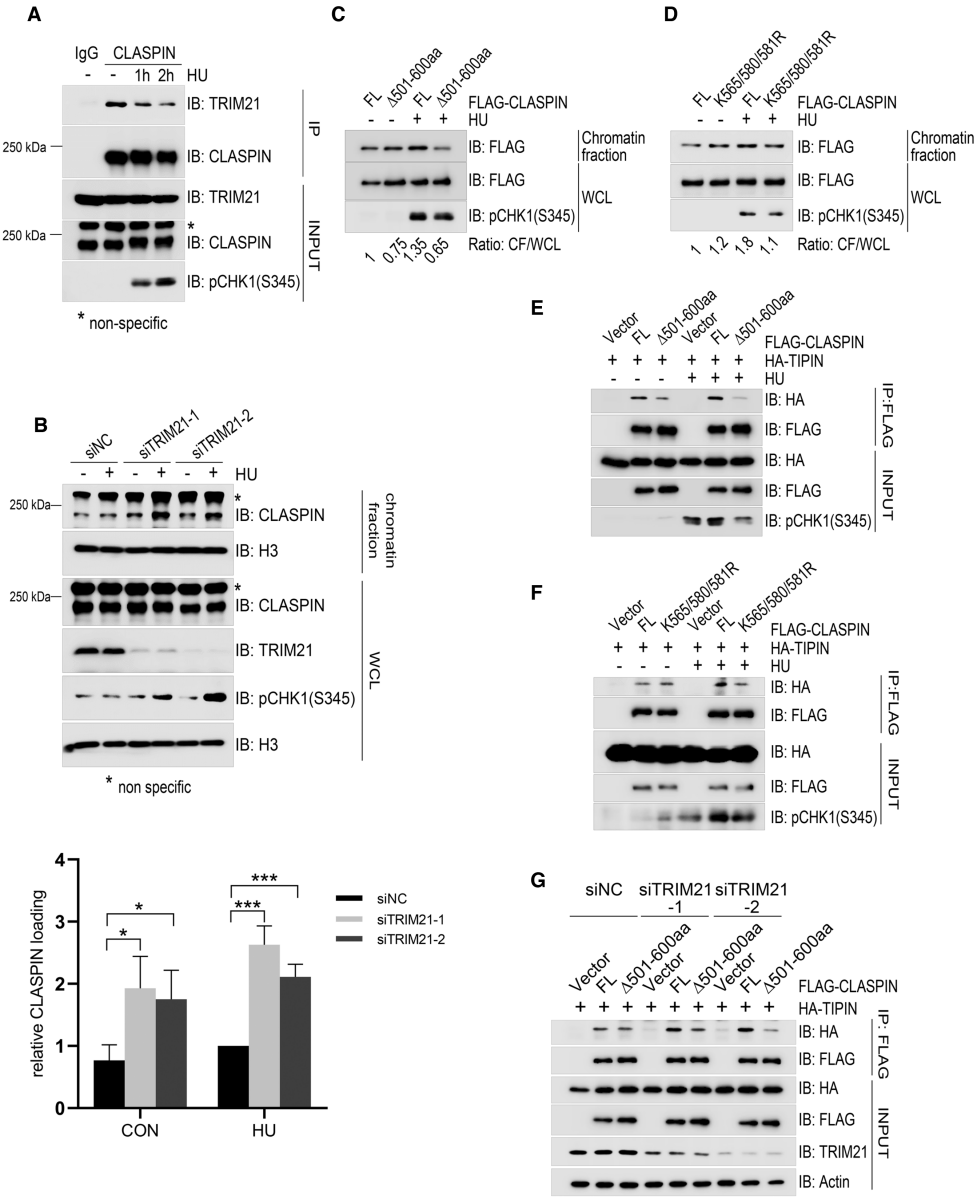
TRIM21 negatively regulated the chromatin loading of CLASPIN and its interaction with TIPIN. (**A**) HEK293T cells were treated with 2 mM HU for the indicated times, total cell lysates were extracted and subjected to immunoprecipitation and immunoblotting with antibodies as indicated. *, non-specific signal. (**B**) HeLa cells were transfected with siNC or siTRIM21 for 24 h and subjected to double thymidine blocks to synchronize cells in G1/S transition followed by a release for 2 h. Transfectants were untreated or treated with 2 mM HU for 1 h before chromatin fractionation was performed. The chromatin-enriched fraction was analyzed by immunoblotting with the indicated antibodies. * Non-specific signal. Statistical analysis (*t*-test) of signal intensity from three independent experiments was shown in the lower panel. *P < 0.05; ***P < 0.001. (**C**) HEK293T cells were transfected with FLAG-CLASPIN or the FLAG-CLASPINΔ501–600 mutant. After 24 h, the cells were treated with 2 mM HU or a mock treatment for 1 h and subjected to chromatin-bound protein extraction. The protein levels of FLAG-CLASPIN or FLAG-CLASPIN**Δ**501–600aa in the chromatin fraction and whole cell lysate were detected by immunoblotting, and relative chromatin loading was analyzed by comparing the ratio between the chromatin fraction and the whole cell lysate. (**D**) HEK293T cells were transfected with FLAG-CLASPIN or the FLAG-CLASPIN K565/580/581R. Then cells were treated and analyzed by immunoblotting as described in (C). (**E**) HEK293T cells were transfected with HA-TIPIN and FLAG-CLASPIN or its Δ501–600aa mutant. After 24 h, the cells were treated with 2 mM HU or a mock treatment for 1 h, and subjected to immunoprecipitation using anti-FLAG M2 agarose, followed by immunoblotting with the indicated antibodies. (**F**) HEK293T cells were transfected with HA-TIPIN and FLAG-CLASPIN or its K565/580/581R mutant. After 24 h, the cells were treated with 2 mM HU or a mock treatment for 1 h and subjected to immunoprecipitation using anti-FLAG M2 agarose, followed by immunoblotting with the indicated antibodies. (**G**) HEK293T cells were transfected with a negative control or different TRIM21 specific siRNAs for 24 h. Then the cells were transfected with HA-TIPIN and FLAG-CLASPIN or its Δ501–600aa mutant, and analyzed by immunoprecipitation using anti-FLAG M2 agarose followed by immunoblotting with the indicated antibodies.

Based on the findings that TRIM21 promotes K63-linked ubiquitination of CLASPIN and counteracts its K6-linked ubiquitination, we reasoned that, while K6-linked ubiquitination of CLASPIN is beneficial for CHK1 activation, its K63-linked ubiquitination impedes this process. As such TRIM21 may function as a negative regulator of CHK1 activation by finetuning the balance of K63/K6-linked ubiquitination of CLASPIN. To test this hypothesis, we first examined the interaction dynamics between TRIM21 and CLASPIN in response to replication stress. Co-immunoprecipitation analysis revealed that the levels of TRIM21 present in the CLASPIN immunocomplex decreased after HU treatment (Figure 4A and Supplementary Figure S8A). We theorized that this dissociation might tip the balance of K63/K6-linked ubiquitination of CLASPIN toward K6-linked ubiquitination. Indeed, HU treatment induced a decrease of K63-linked ubiquitination of CLASPIN (Supplementary Figure S8B) and concurrently an increase of K6-linked ubiquitination of CLASPIN (Supplementary Figure S8C). We further tested if alteration of TRIM21 expression levels changed the cell cycle distribution. Cell cycle profile analysis revealed that S phase distribution in TRIM21-depleted cells or TRIM21-overexpressing cells was similar to that in the wildtype cells (Supplementary Figure S9A and S9B), indicating that TRIM21 suppressed CHK1 activation by regulating the ubiquitination of CLASPIN, but not by affecting the cell cycle profiles.

As CLASPIN mediates ATR-dependent CHK1 activation on chromatin, we next examined if TRIM21-regulated ubiquitination affected the accumulation of CLASPIN on chromatin. It was found that, in S phase synchronized cells, inhibition of TRIM21 expression by two independent siRNA oligos led to an increase of CLASPIN in the chromatin-enriched fraction under unperturbed conditions and a further increase upon HU treatment in comparison to the siRNA control (Figure 4B and Supplementary Figure S9C). Furthermore, when we expressed the K63/K6-linked ubiquitination defective mutants FLAG-CLASPINΔ501–600aa or FLAG-CLASPIN K565/580/581R in HEK293T cells, we failed to observed any obvious increase of FLAG-CLASPINΔ501–600aa or FLAG-CLASPIN K565/580/581R in the chromatin-enriched fraction after HU treatment (Figure 4C and D). These findings indicated that TRIM21 represses CLASPIN chromatin loading, and that K63/K6-linked ubiquitination capacity is a requisite for CLASPIN chromatin loading.

**Figure 5.**
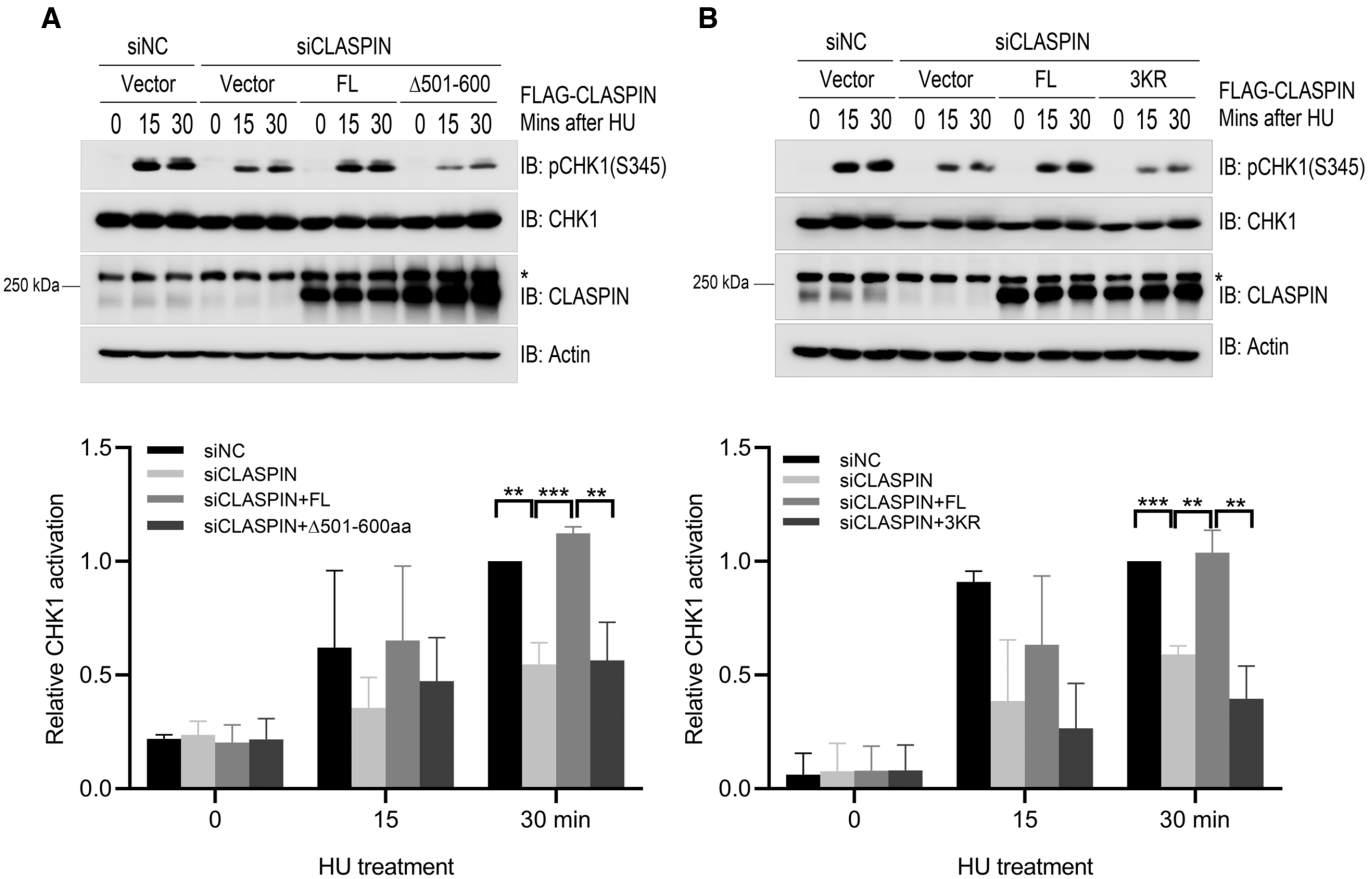
TRIM21-mediated CLASPIN ubiquitination suppressed CHK1 activation. (**A**) HEK293T cells were transfected with a negative control or a siRNA targeting for the 3’UTR of CLASPIN (siCLASPIN) for 24 h. Then, the cells were transfected with FLAG-CLASPIN or FLAG-CLASPINΔ501–600aa as indicated and incubated for a further 24 h. Then the cells were treated with 2 mM HU or a mock treatment before the whole cell lysates were collected and analyzed by immunoblotting. Activation of CHK1 was examined by immunoblotting with an antibody specifically recognizing phosphorylated CHK1 at Ser345. The CHK1 and CLASPIN expression levels were also examined. Actin: loading control. *, non-specific signal. Statistical analysis (*t*-test) of signal intensity from three independent experiments was shown in the lower panel. **P < 0.01; ***P < 0.001. (**B**) HEK293T cells were transfected with a negative control or a CLASPIN-specific siRNA for 24 h. Then, the cells were transfected with FLAG-CLASPIN or FLAG-CLASPIN K565/580/581R (3KR) mutant as indicated, and incubated for a further 24 h. Then the cells were treated and examined as in (A). *, non-specific signal. Statistical analysis (*t*-test) of signal intensity from three independent experiments was shown in the lower panel. **P < 0.01; ***P < 0.001.

TIPIN (TIMELESS-interacting protein) interacts with RPA (RPA2), which stabilizes both the TIMELESS-TIPIN complex and CLASPIN on RPA-coated ssDNA and facilitates ATR-dependent CHK1 activation (10). We thus explored how TRIM21 impacts on the CLASPIN-TIPIN interaction and subsequent CHK1 activation. Co-immunoprecipitation analysis found that FLAG-CLASPIN interacted with HA-TIPIN under unperturbed conditions and this interaction was enhanced in response to HU treatment (Figure 4E and F), while the interaction between CLASPIN ubiquitination defective mutants FLAG-CLASPINΔ501–600aa or K565/580/581R and HA-TIPIN were compromised upon HU treatment (Figure 4E and F). Inhibiting TRIM21 expression increased the interaction between FLAG-CLASPIN and HA-TIPIN (Figure 4G and Supplementary Figure S10A), while only minimally affected the interaction between FLAG-CLASPINΔ501–600aa and HA-TIPIN (Figure 4G). Also, the interaction between FLAG-CLASPIN and endogenous CHK1 was increased in TRIM21-depleted cells (Supplementary Figure S10B). Furthermore, we found that inhibition of CLASPIN expression by siRNA compromised HU-induced CHK1 activation, while re-expression of CLASPIN, but not the TIPIN/CLASPIN interaction defective mutant FLAG-CLASPINΔ501–600aa or K565/580/581R, rescued CHK1 activation (Figure 5A and B). Also, under prolonged replication stress, CLASPIN-depleted cells with re-expression of FLAG-CLASPINΔ501–600aa or K565/580/581R compromised CHK1 activation (Supplementary Figure S11A and S11B), and this compromise was rescued by overexpression of HA-TIPIN (Supplementary Figure S11C and S11D). Together, these results demonstrate that TRIM21 negatively regulates chromatin loading of CLASPIN by interfering with the interaction between TIPIN and CLASPIN.

### Overexpression of TRIM21 leads to instability of stalled replication forks

**Figure 6.**
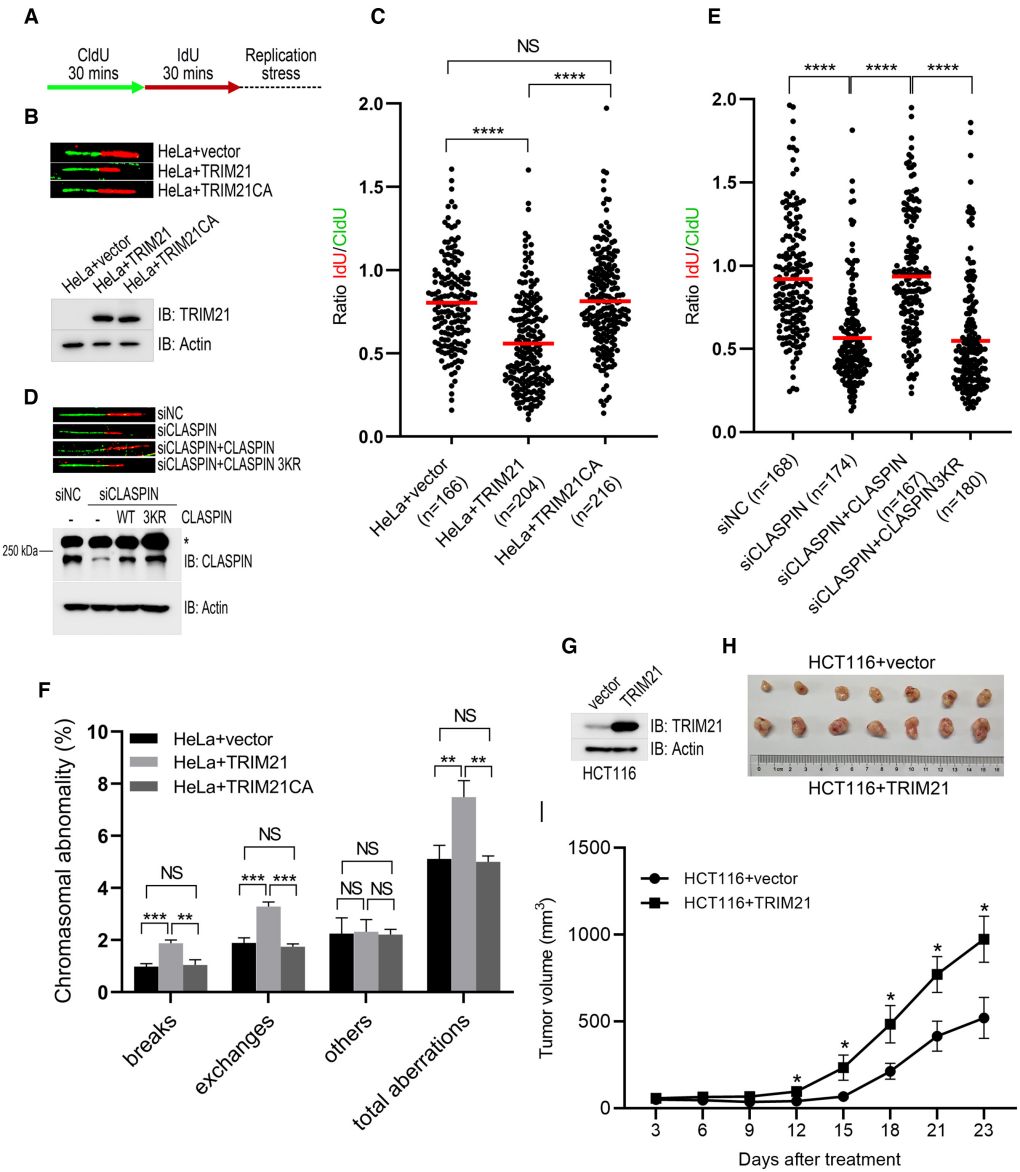
Overexpression of TRIM21 led to genome instability and tumorigenesis. (**A**) Schematic of the DNA fiber assay examining stalled replication fork stability. Cells were sequentially labeled with 40 μM CldU and 100 μM IdU for 30 min each, followed by treatment with 5 mM hydroxyurea and 5 μM aphidicolin to block replication fork progression. (B, C) HeLa cells or HeLa cells overexpressing either TRIM21 or TRIM21(CA) were treated as described in (A); the DNA fibers were spread and subjected to immunofluorescence using anti-BrdU antibodies specifically recognizing CldU or IdU. (**B**) Representative images of the DNA fibers and the TRIM21 expression level. (**C**) For each group, a IdU/CldU ratio of > 150 individual DNA fibers is presented and the mean IdU/CldU ratio (marked as the red line) is shown. ^****^, *t*-test, *P* < 0.0001. (D, E) HeLa cells and HeLa cells expressing exogenous CLASPIN or ubiquitination-defective mutant CLASPIN(K565/580/581R) were transfected with siNC or siCLASPIN for 48 h before subjected to DNA fiber assay as described in (A) and immunoblotting with antibodies as indicated. Representative images of the DNA fibers and the CLASPIN expression levels are shown in (**D**). * Non-specific signal. (**E**) For each group, a IdU/CldU ratio of >150 individual DNA fibers is presented and the mean IdU/CldU ratio (marked as the red line) are shown in (E). ^****^, *t*-test, *P* < 0.0001. (**F**) HeLa cells stably overexpressing TRIM21 or its E3 ligase inactive mutant were subjected to metaphase spread assay; >1500 metaphase chromosomes were analyzed for every cell line. The error bars represent the SD, *n* = 3. *t*-test, **P < 0.01; ***P < 0.001. (**G–I**) 6 × 10^6^ HCT116 cells overexpressing empty vector or TRIM21 were implanted into nude mice, and the tumor volume was monitored at the indicated times. The TRIM21 expression level was examined (G). Tumor images (H) and quantification results are shown (I). *n* = 7, mean tumor volume ± SEM, *t*-test, *P < 0.05.

Stalled replication forks are vulnerable and predisposed to collapse if not stabilized, resulting in the generation of DSBs and genome instability, which is a key driver leading to tumorigenesis (3). CHK1 activation is essential for optimized fork stabilization under replication stress (46,47). As TRIM21 negatively regulated CHK1 activation upon replication stress, we reasoned that overexpression of TRIM21 would result in instability of stalled replication forks and potentially promote tumorigenesis. To test these hypotheses, we performed DNA fiber assays in HeLa cells stably overexpressing TRIM21 or TRIM21 CA. We sequentially labeled these cells with the thymidine analogues CldU and IdU for 30 min and then treated the cells with HU and APH (aphidicolin) to block replication fork progression (Figure 6A). CldU and IdU signal would exhibit equal length as the same labeling duration, and a shortened IdU signal reflects the unstable replication fork. We found that over-expression of TRIM21, but not the catalytically inactively mutant TRIM21 CA, caused a significant reduction of IdU/CldU ratio (Figure 6B and C), indicating that TRIM21 overexpression renders newly synthesized DNA at the stalled replication forks susceptible to degradation. Conversely, newly synthesized DNA at the stalled replication forks were more stable in TRIM21-depleted cells comparing with that in wildtype HeLa cells (Supplementary Figure S12A and S12B). We further found that CLASPIN deficiency led to a significant reduction of IdU/CldU ratio, and re-expression of wildtype CLASPIN, but not its ubiquitination defective mutant K565/580/581R, efficiently restored stalled replication fork stability (Figure 6D and E). Metaphase spread assay showed that overexpression of TRIM21, but not the catalytically inactively mutant TRIM21 CA, resulted in increased aberrant chromosomes (Figure 6F and Supplementary Figure S13). Finally, HCT116 cells stably overexpressing TRIM21 xenografted onto nude mice developed significantly larger tumor volumes than control HCT116 cells (Figure 6G–I). These results suggest that TRIM21 overexpression results in instability of stalled replication fork and promotes tumorigenesis.

## DISCUSSION

CLASPIN is a critical player in checkpoint responses and replication fork stabilization. RPA-bound single strand DNA efficiently triggers the ATR-CHK1 pathway to initiate the cell cycle checkpoint and stabilizes stalled replication forks to safeguard genome stability (6,7). CLASPIN exhibits dynamic expression levels throughout the cell cycle, and is recruited to stalled replication forks by TIPIN and undergoes phosphorylation in ATR-dependent manner; phosphorylated CLASPIN in turn interacts with and presents CHK1 to ATR for phosphorylation and activation (10–12). Timely switching on/off of CHK1 could be accomplished through protein turnover and stability of CLASPIN, which is strictly regulated by a series of ubiquitin ligases and deubiquitinases (14–22).

In addition to proteasome-mediated degradation, non-degradation related ubiquitination of CLASPIN also plays roles in regulating CHK1 activation: BRCA1 is reported to mediate ubiquitination of CLASPIN on the N-terminal region (41–101aa), and this modification is not responsible for the turnover of CLASPIN but promotes its chromatin loading and subsequent CHK1 activation upon DNA replication stress (23).

In this study, we constructed a series of deletion mutants in the context of full-length CLASPIN (Figure 2C) to determine the essential regions of K63/K6 linked ubiquitination of CLASPIN. Surprisingly, ΔF3 (601–930 aa) mutant exhibits much stronger ubiquitination with K6/K63 linkage (Figure 3E and H). Given that this mutant efficiently interacts with TRIM21 (Figure 2D) and TRIM21-mediated K63 ubiquitination antagonizes K6-ubiquitination of CLASPIN, we reason that this region may not have any impact on TRIM21 enzymatic activity and speculate that this region may potentially promote recruitment of certain deubiquitinases essential for CLASPIN deubiquitination.

We further show that TRIM21-mediated K63-linked ubiquitination of CLASPIN counteracts its K6-linked ubiquitination on 501–600aa, leading to its dissociation from TIPIN and prevention of CHK1 activation under replication stress. CLASPIN is overall negatively charged but contains two basic residue-enriched regions distributed in the N-terminus which are responsible for interacting with replication forks (48). 501–600aa locates in the basic patch (BP II, 492–622aa) of CLASPIN, which is enriched with lysine residues. Here, we show that TRIM21-mediated K63-linked ubiquitination of CLASPIN mainly takes place on 501–600aa that contains 13 lysine residues, we examined the K63-linked and K6-linked ubiquitination of every single point mutant among all these 13 lysine residues (Supplementary Figure S7E, S7F). We detected compromised K6-linked and K63-linked ubiquitination on K565R, K580R and K581R mutants and greatly reduced ubiquitination on the triple mutant. We thus believe that K565/580/581 are the major sites for K6/63 ubiquitination in CLASPIN. Ubiquitination with K63-linkage or K6-linkage on this domain competes for lysine residues and regulates CHK1 activation. K6-linked ubiquitination on 501–600aa of CLASPIN seems necessary for its interaction with TIPIN and is beneficial for CHK1 activation (Figures 4E, 5A, and Supplementary Figure S11A). Deletion of 501–600aa or mutation of K565/580/581 residues responsible for K6/K63-linked ubiquitination removes CLASPIN’s capacity to promote CHK1 activation (Figures 3G, I–K, 5A-B, and Supplementary Figure S11A-S11D). Depletion of TRIM21 promotes the interaction between TIPIN and CLASPIN; however, TRIM21 minimally regulates the interaction between TIPIN and CLASPIN mutant without the 501–600 aa region (Figure 4G). Thus, a dynamic association between TRIM21 and CLASPIN ensures appropriate CHK1 activation. In unperturbed conditions, TRIM21 associates with CLASPIN and mediates its K63-linked ubiquitination, which counteracts its K6-linked ubiquitination; while under replication stress, TRIM21 dissociates from CLASPIN, and decreased ubiquitination with K63-linkage and increased ubiquitination with K6-linkage on CLASPIN thereby promotes CHK1 activation (Figure 4A and Supplementary Figure S8A–S8C).

TRIM21 has an essential role as a negative regulator of innate immune responses by targeting DDX41, IRF3, IRF5, and other interferon response factors for proteasomal degradation(49–53). Emerging evidence demonstrated that TRIM21 also participates in the modulation of DNA damage response by indirectly suppressing p53 stability or synthesis via GMPS and HuR (31,54,55). In this study, we demonstrate that in response to replication stress, over-expression of TRIM21 in cancer cells ubiquitinates CLASPIN with K63-linkage type, which counteracts its K6-linked ubiquitination, thus compromising CHK1 activation and stability of stalled replication forks.

It is not conclusive yet if TRIM21 plays a pro-oncogenic role or a suppressive role during tumorigenesis; some studies show that TRIM21 is upregulated in a broad spectrum of cancers and promotes the proliferation of breast cancer cell lines (31), and it is upregulated in gliomas and negatively correlated with the prognosis of glioma patients(32). Conversely, TRIM21 is downregulated in colorectal cancer and negatively regulates intestinal epithelial carcinogenesis(56), although interestingly downregulation of TRIM21 is also found to promote carcinogenesis and indicate poor prognosis of hepatocellular carcinoma and breast cancer(57,58). According to the combined analysis of TCGA and GTEx data, TRIM21 is upregulated in a wide spectrum of cancers, such as glioma, melanoma, pancreatic adenocarcinoma and colorectum adenocarcinoma (Supplementary Figure S1). Our results also show that upregulation of TRIM21 in HCT116 colorectal cancer cells promotes tumorigenesis in xenografted mice (Figure 6G–I).

Overall, we propose that TRIM21 has a pro-oncogenic role in tumorigenesis at least in part through suppressing CHK1 activation in response to endogenous replication stress and stability of stalled replication forks. The result of this cascade is genomic instability and ultimately tumorigenesis. Our findings imply that TRIM21 catalytic activity could be a potential target for anti-cancer drug development and therapy.

## DATA AVAILABILITY

GEPIA is a web server for cancer and normal gene expression profiling and interactive analyses, and available at http://gepia.cancer-pku.cn/.

## Supplementary Material

gkac011_Supplemental_File
