# Supplementary material for: TRIM21 suppresses CHK1 activation by preferentially targeting CLASPIN for K63-linked ubiquitination
Source: Nucleic Acids Res. 2022 Jan 20;50(3):1517–30. doi: 10.1093/nar/gkac011 (PMC8860585; doi:10.1093/nar/gkac011)
Supplement: gkac011_Supplemental_File [file gkac011_supplemental_file.pdf]

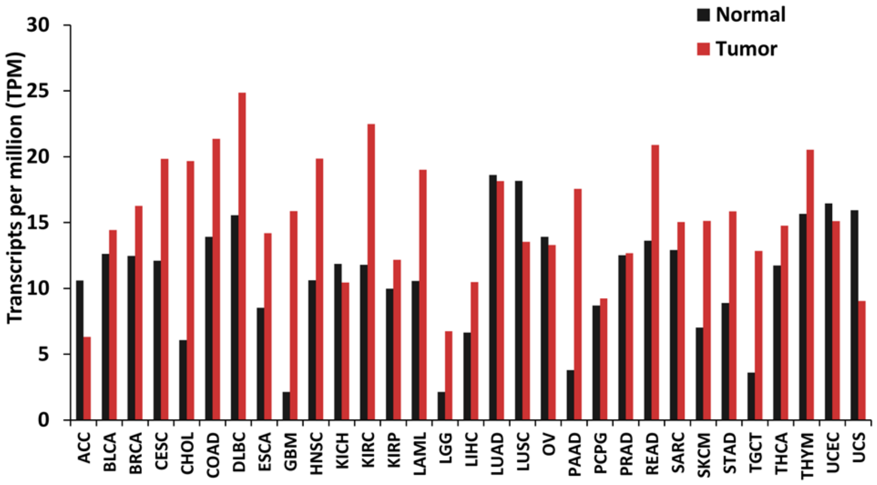

Supplementary Figure 1. TRIM21 expression profile across different tumor samples and paired normal tissues. This profile is from the GEPIA web server (http://gepia.cancer-pku.cn/detail.php) that analyzes RNA sequencing expression data based upon data from the TCGA (the Cancer Genome Atlas) program and GTEx (Genotype-Tissue Expression) project. This expression profile was obtained by searching TRIM21 in the General tab on GEPIA website.

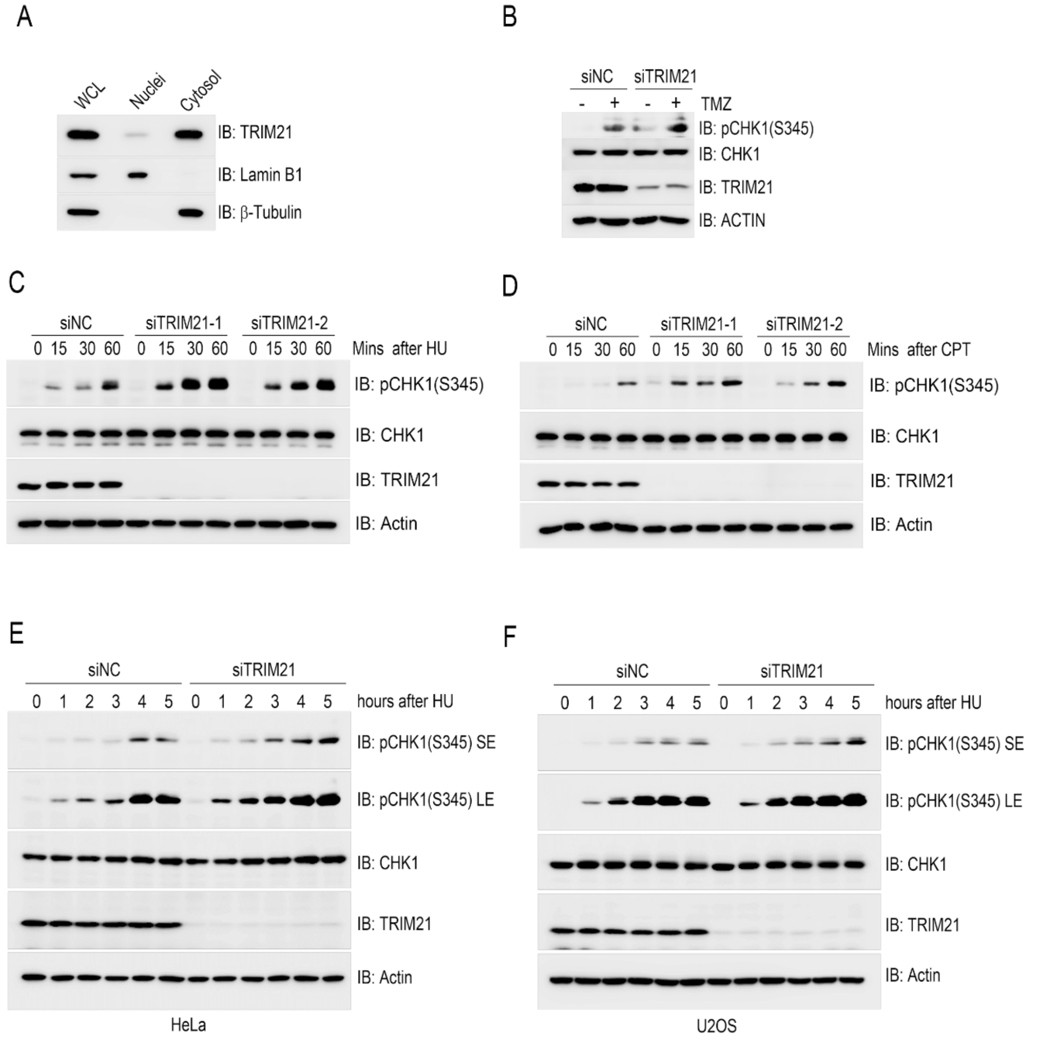

Supplementary Figure 2 (related to Figure 1). TRIM21 suppressed CHK1 activation upon replication stress. (A) Subcellular localization of TRIM21. Nuclei fraction of HeLa cells were extracted as described in "materials and methods" and analyzed by immunoblotting with antibodies as indicated. **(B)** U87 cells were transfected with a negative control siRNA or siRNA targeting TRIM21. After 48 hours, the whole cell lysates were collected after a treatment with 200 μM temozolomide or a mock treatment for 2 h. CHK1 activation was examined by immunoblotting with an antibody specifically recognizing phosphorylated CHK1 at Ser345. The CHK1 and TRIM21 expression levels were also examined. Actin: loading control. **(C)** U2OS cells were transfected with a negative control siRNA (siNC) or siRNA targeting 3'UTR of TRIM21 (siTRIM21-1 and siTRIM21-2) for 48 h. Transfectants were treated with 2 mM HU for the indicated time and total cell lysates were harvested for immunoblotting with antibodies as indicated. **(D)** U2OS cells were treated as described in (C) except that HU

treatment was replaced with CPT (1 μM) treatment. **(E)** HeLa cells were transfected with siNC or siTRIM21 for 48 h followed by a treatment with 2 mM HU or a mock treatment for indicated time. Total cell lysates were extracted and subjected to immunoblotting with antibodies as indicated. **(F)** U2OS cells were treated and processed as described in (E).

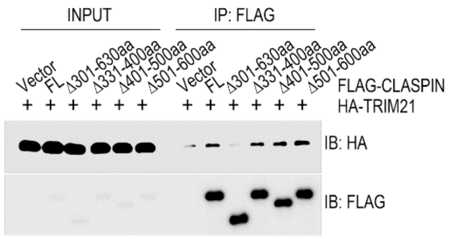

Supplementary Figure 3 (related to Figure 2). TRIM21 interacted with CLASPIN. HEK293T cells were co-transfected with HA-TRIM21 and wildtype or mutant FLAG-CLASPIN. Whole cell lysates were incubated with anti-FLAG M2 agarose before immunoprecipitation, followed by immunoblotting with the indicated antibodies.

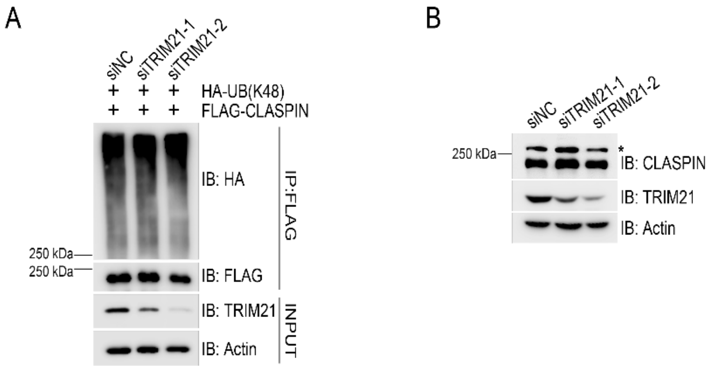

Supplementary Figure 4 (related to Figure 3). TRIM21 didn't affect the stability of CLASPIN. (A) HEK293T cells were transfected with a negative control or TRIM21-specific siRNA for 24 h. Then the cells were co-transfected with FLAG-CLASPIN and HA-ubiquitin K48-only plasmids. After 24 h, all the cell lines were lysed and subjected to de-naturing immunoprecipitation using anti-FLAG M2 agarose. The immunoprecipates were examined by immunoblotting with the indicated antibodies. **(B)** Whole cell lysates were extracted from 293T

cells transfected with a negative control or TRIM21-specific siRNA and then analyzed by immunoblotting with the indicated antibodies. * non-specific signal.

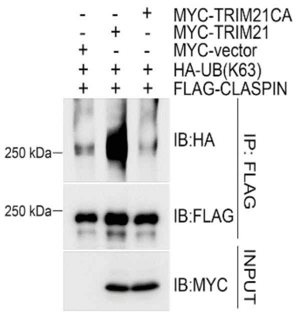

Supplementary figure 5 (related to Figure 3). Overexpression of TRIM21 promoted K63-linked ubiquitination of CLASPIN. HEK293T cells were transfected with FLAG-CLASPIN and HA-ubiquitin K63-only (HA-UB(K63)) together with MYC-TRIM21 or the catalytically inactive mutant MYC-TRIM21CA as indicated. After 24 h, total cell lysates were extracted under denaturing conditions and subjected to immunoprecipitation using anti-FLAG M2 agarose. The immunoprecipitated complexes were examined by immunoblotting with antibodies as indicated.

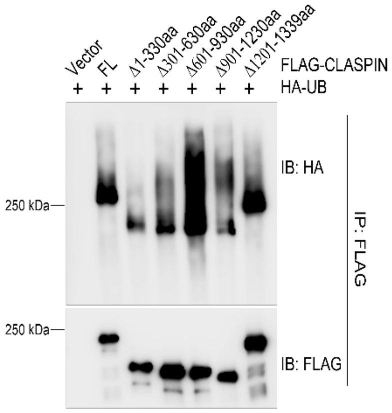

Supplementary figure 6 (related to Figure 3). Total ubiquitination level of different CLASPIN mutants. HEK293T cells co-transfected with HA-ubiquitin and wildtype or mutant FLAG-CLASPIN were lysed and subjected to de-naturing immunoprecipitation using anti-FLAG M2 agarose. The immunoprecipitates were examined by immunoblotting with the indicated antibodies.

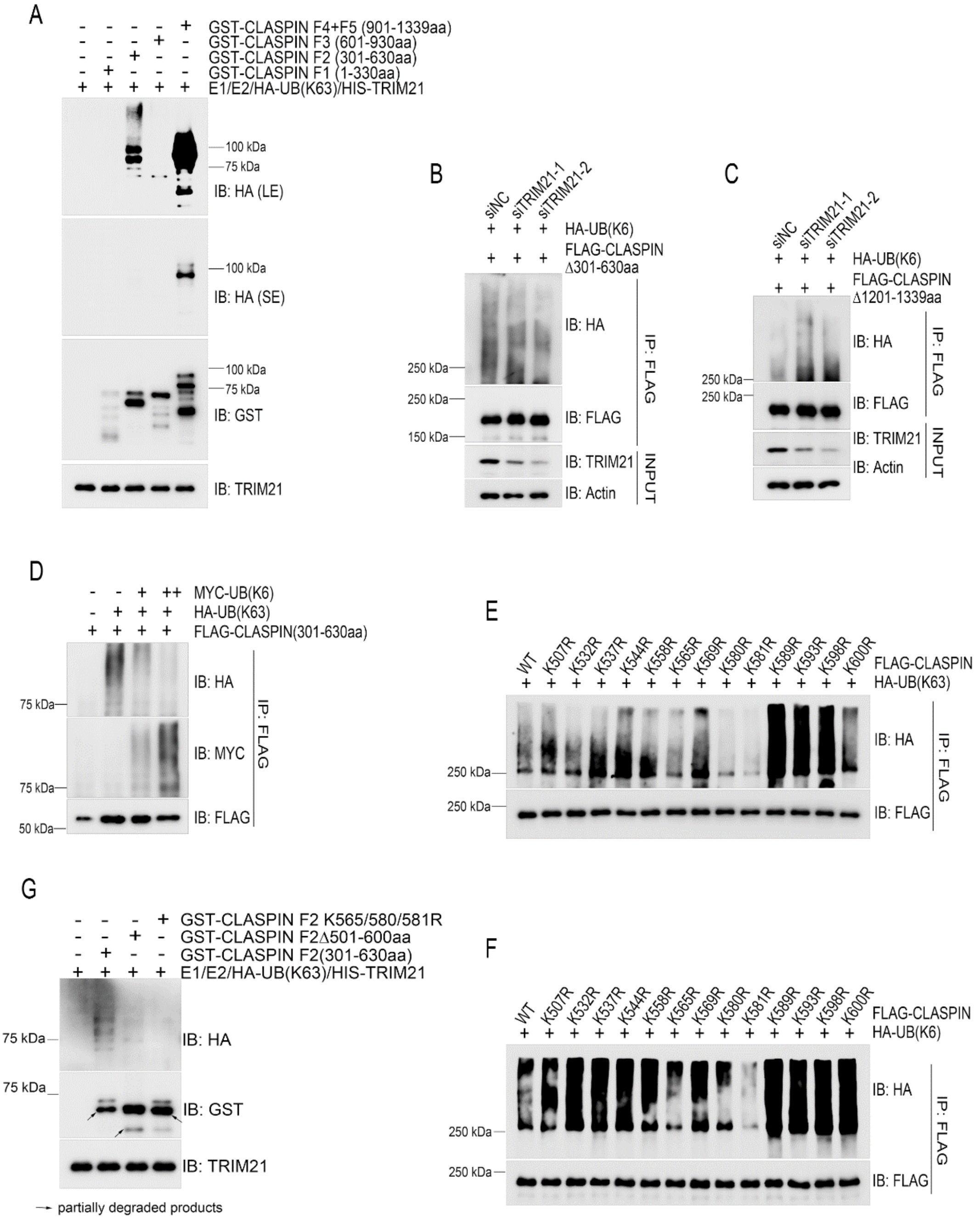

Supplementary Figure 7 (related to Figure 3). K6-linked ubiquitination of CLASPIN regulated by TRIM21 fell in the region of 301-630aa but not 1201-1339aa. (A) *In vitro* ubiquitination assays were performed by incubating HIS-TRIM21 with GST-CLASPIN F1(1-330aa) or F2 (301-630aa) or F3 (601-930aa) or F4+F5 (901-1339aa) fragment in the presence of E1, E2 and HA-ubiquitin K63-only (HA-UB(K63)) at 30℃ for 1 h, followed by GST-pulldown and immunoblotting with antibodies as indicated. **(B-C)** HEK293T cells were transfected with a negative control or different TRIM21-specific siRNAs. After 24 h, the cells

were co-transfected with HA-ubiquitin K6-only and FLAG-CLASPINΔ301-630aa (as in **B**) or FLAG-CLASPINΔ1201-1339aa (as in **C**). After a further 24 h, all the cell lines were lysed and subjected to de-naturing immunoprecipitation and immunoblotting with the indicated antibodies. **(D)** HEK293T cells were transfected with the FLAG-CLASPIN F2 (301-630aa) fragment and HA-ubiquitin K63-only along with increasing doses of MYC-ubiquitin K6-only expressing plasmids. After 24 h, the cells were lysed and subjected to de-naturing immunoprecipitation using anti-FLAG M2 agarose, followed by immunoblotting with the indicated antibodies. **(E-F)** HEK293T cells were co-transfected with HA-UB(K63) (as in **E**) or HA-UB(K6) (as in **F**) and wildtype FLAG-CLASPIN or its KR mutants. The samples were analyzed by de-naturing immunoprecipitation and immunoblotting with the indicated antibodies. **(G)** *In vitro* ubiquitination assays were performed by incubating HIS-TRIM21 with GST-CLASPIN F2 (301-630aa) or CLASPIN F2(Δ501-600aa) or CLASPIN F2(K565/580/581R) in the presence of E1, E2 and HA-UB(K63) at 30℃ for 1 h, followed by GST-pulldown and analysis by immunoblotting with the indicated antibodies.

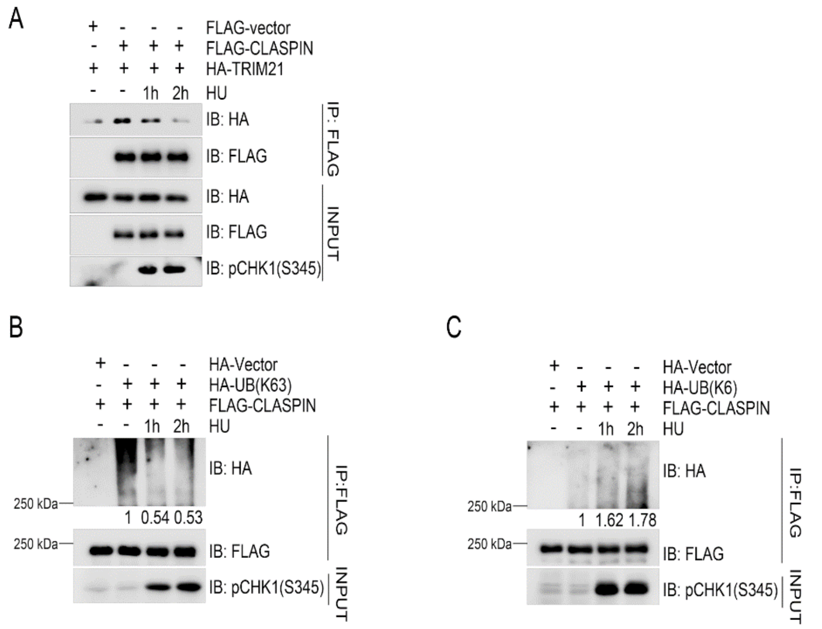

Supplementary Figure 8 (related to Figure 4). Dynamic of K6-linked and K63-linked ubiquitination of CLASPIN in response to replication stress. (A) HEK293T cells co-expressing FLAG-CLASPIN and HA-TRIM21 were treated with 2 mM HU for the indicated

times, total cell lysates were extracted and subjected to immunoprecipitation and immunoblotting with antibodies as indicated. **(B-C)** HEK293T was transfected with FLAG-CLASPIN and HA-ubiquitin K63-only (as in **B**) or HA-ubiquitin K6-only (as in **C**) for 24 h. Then, the cells were treated with 2 mM HU or a mock treatment for indicated time before de-naturing immunoprecipitation using anti-FLAG M2 agarose. The immunoprecipitates were analyzed by immunoblotting with the indicated antibodies.

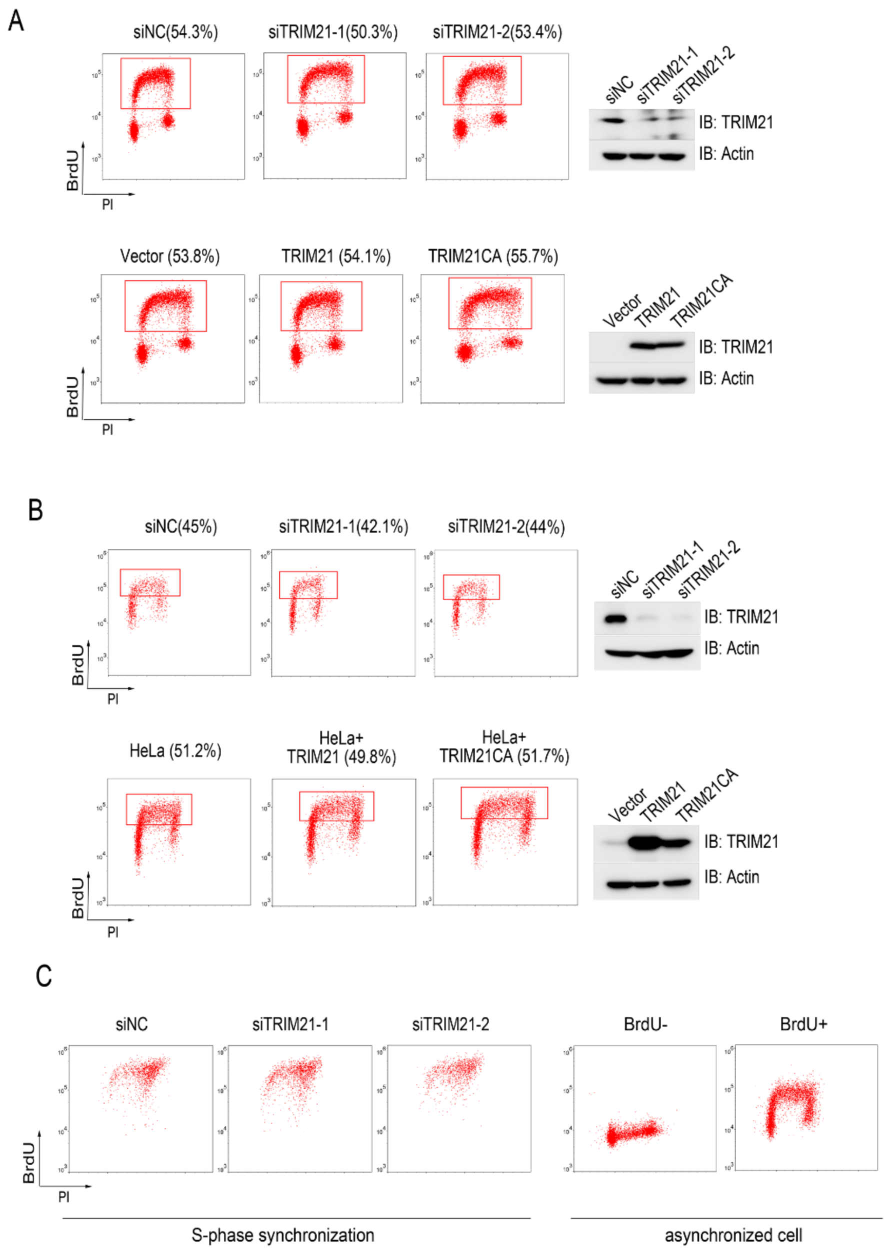

Supplementary Figure 9 (related to Figure 4). Alteration of TRIM21 expression levels didn't have an impact on the cell cycle progression. (A) HEK293T cells were transfected with siNC or siTRIM21 for 48 h, or transfected with TRIM21 or TRIM21(CA) for 24 h. Transfectants were pulse labeled with BrdU for 30 min, followed by BrdU and PI staining and flow cytometric analysis. Percentage within a parenthesis represents BrdU-positive cells. **(B)** HeLa cells transfected with siNC or siTRIM21 or overexpressing TRIM21 or TRIM21(CA) were treated and processed as described in (A). **(C)** HeLa cells were transfected with siNC or siTRIM21 for 24 h and subjected to double thymidine blocks to synchronize cells in G1/S transition followed by a release for 2 h. Transfectants then were pulse labeled with BrdU for 30 min, followed by BrdU and PI staining and flow cytometric analysis. Flow cytometric analysis of asynchronized HeLa cells with or without BrdU labeling were also presented.

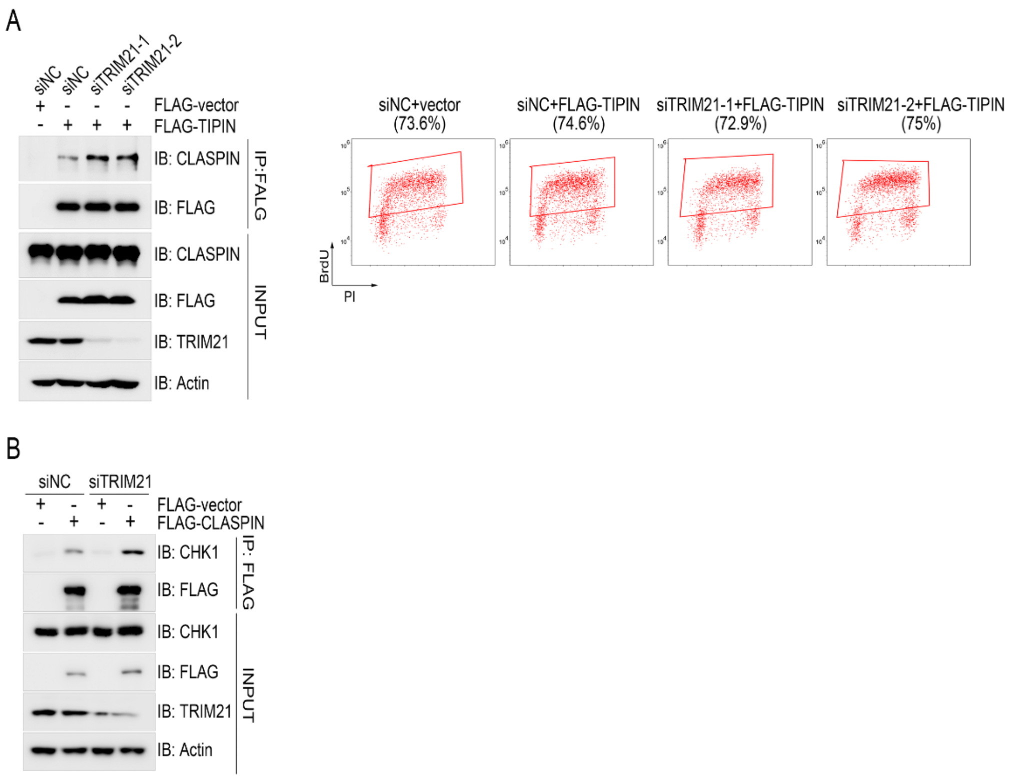

Supplementary Figure 10 (related to Figure 4). TRIM21 negatively regulated the interaction between CLASPIN and TIPIN or CHK1. (A) Depletion of TRIM21 promoted the interaction between CLASPIN and TIPIN. HeLa cells were transfected with siNC or siTRIM21 for 24 h followed by transfection with FLAG-TIPIN for 8 h and subjected to double thymidine

blocks to synchronize cells at G1/S boundary. Transfectants after release for 2 h were pulse labeled with BrdU for 30 min. Then cells were lysed and incubated with anti-FLAG M2 agarose, the immunoprecipitates were then examined by immunoblotting with antibodies as indicated. Flow cytometric analysis of BrdU and PI staining were also presented. **(B)** Depletion of TRIM21 promoted the interaction between CLASPIN and CHK1. HEK293T cells were transfected with siNC or siTRIM21 for 24 h followed by transfection with FLAG-CLASPIN. Total cell lysates were extracted 24 h later and subjected to immunoprecipitation with anti-FLAG M2 agarose and immunoblotting with antibodies as indicated.

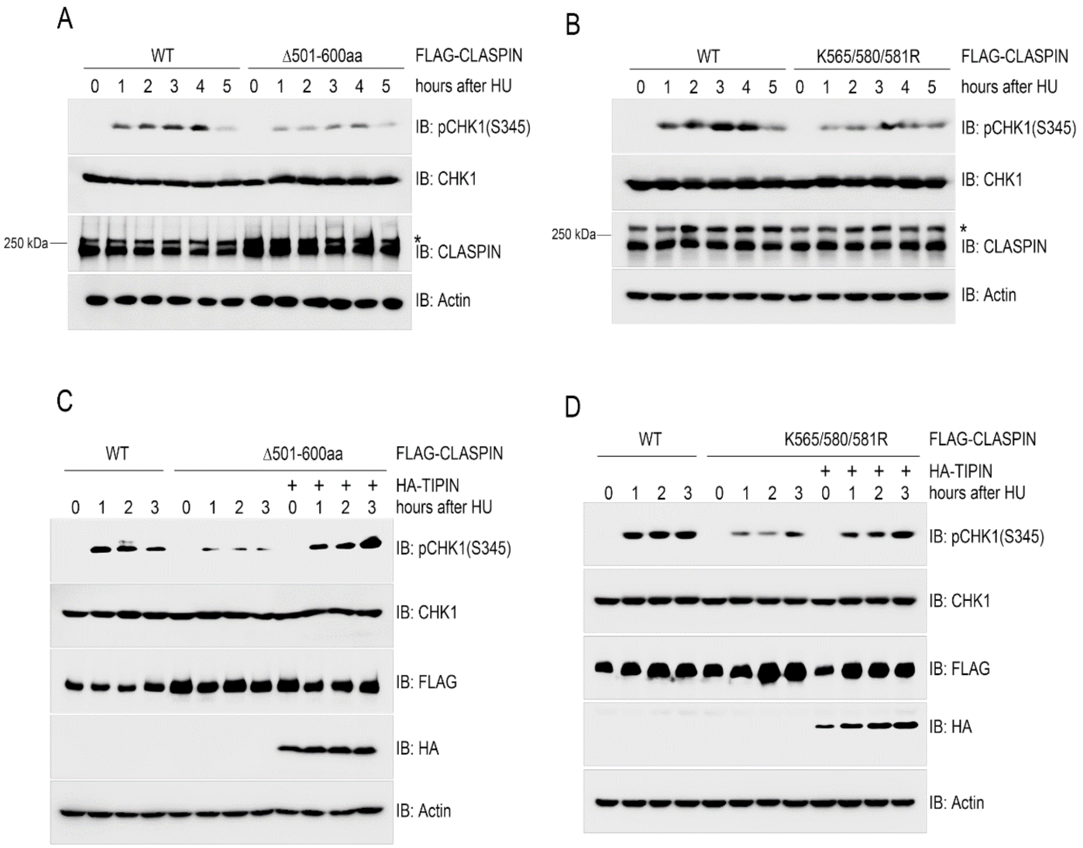

Supplementary Figure 11 (related to Figure 5). Expression of CLASPIN(Δ501-600aa) or CLASPIN(K565/580/581R) compromises replication stress-induced CHK1 activation. (A and B) CLASPIN-depleted HEK293T cells were transfected with FLAG-CLASPIN or FLAG-CLASPINΔ501-600aa **(A)** or FLAG-CLASPIN(K565/580/581R) **(B)** for 24 h. Transfectants then were treated with 2 mM HU for indicated time before total cell lysates were extracted and subjected to immunoblotting with antibodies as indicated. *, non-specific signal. **(C)** CLASPIN-

depleted HEK293T cells were transfected with FLAG-CLASPIN or FLAG-CLASPINΔ501-600aa, or co-transfected with FLAG-CLASPINΔ501-600aa mutant and HA-TIPIN for 24 h. Transfectants then were treated with 2 mM HU for indicated time before total cell lysates were extracted and subjected to immunoblotting with antibodies as indicated. **(D)** CLASPIN-depleted HEK293T cells were treated and processed as described in (C) except FLAG-CLASPINΔ501-600aa was replaced with FLAG-CLASPIN(K565/580/581R).

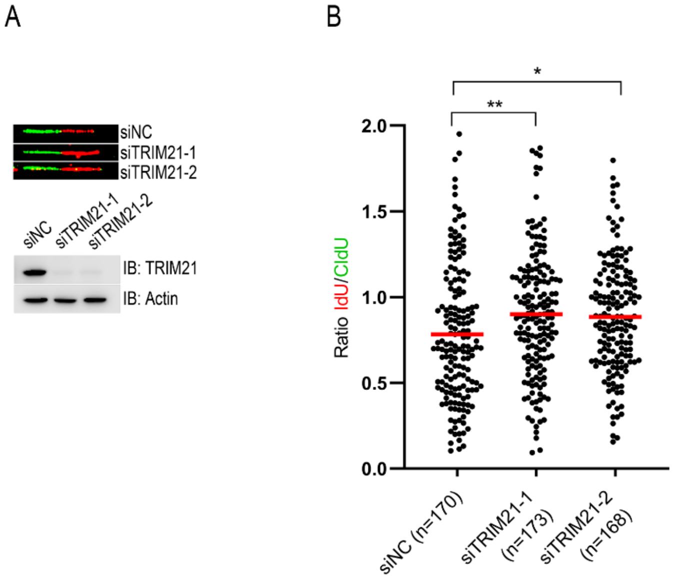

Supplementary Figure 12 (related to Figure 6). Depletion of TRIM21 is beneficial for stalled replication fork stability. HeLa cells were transfected with siNC or siTRIM21 for 48 h before subjected to DNA fiber assay as described in Figure 6A and immunoblotting with antibodies as indicated. Representative images of the DNA fibers and the TRIM21 expression levels are shown in (A). For each group, a IdU/CldU ratio of >150 individual DNA fibers is presented and the mean IdU/CldU ratio (marked as the red line) are shown in (B). *, *t*-test, P<0.05; **, P<0.01.

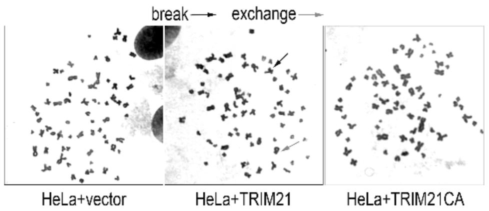

Supplementary Figure 13 (related to Figure 6). Representative image of metaphase spread. HeLa cells stably overexpressing empty vector, TRIM21 or TRIM21CA mutant were used for metaphase spread preparation. Cells were treated with 0.4 μg/mL colchicine for 4 h before being harvested. The chromosomal spreads were prepared after hypotonic treatment with 0.056M KCl and fixation with methanol/acetic acid (volume ratio of 3:1), and stained with Giemsa before images were captured.
